# Bread-Derived Bioactive Porous Scaffolds: An Innovative and Sustainable Approach to Bone Tissue Engineering

**DOI:** 10.3390/molecules24162954

**Published:** 2019-08-14

**Authors:** Elisa Fiume, Gianpaolo Serino, Cristina Bignardi, Enrica Verné, Francesco Baino

**Affiliations:** 1 Institute of Materials Physics and Engineering, Department of Applied Science and Technology (DISAT), Politecnico di Torino, Corso duca degli Abruzzi 24, 10129 Torino, Italy; 2 Department of Mechanical and Aerospace Engineering (DIMEAS), Politecnico di Torino, Corso Duca degli Abruzzi 24, 10129 Torino, Italy

**Keywords:** bioactive glass, scaffold, template replication, porous biomaterials, sustainable materials, bone tissue engineering

## Abstract

In recent years, bioactive glasses gained increasing scientific interest in bone tissue engineering due to their capability to chemically bond with the host tissue and to induce osteogenesis. As a result, several efforts have been addressed to use bioactive glasses in the production of three-dimensional (3D) porous scaffolds for bone regeneration. In this work, we creatively combine typical concepts of porous glass processing with those of waste management and propose, for the first time, the use of bread as a new sacrificial template for the fabrication of bioactive scaffolds. Preliminary SEM investigations performed on stale bread from industrial wastes revealed a suitable morphology characterized by an open-cell 3D architecture, which is potentially able to allow tissue ingrowth and vascularization. Morphological features, mechanical performances and in vitro bioactivity tests were performed in order to evaluate the properties of these new “sustainable” scaffolds for bone replacement and regeneration. Scaffolds with total porosity ranging from 70 to 85 vol% and mechanical strength comparable to cancellous bone were obtained. Globular hydroxyapatite was observed to form on the surface of the scaffolds after just 48-h immersion in simulated body fluid. The results show great promise and suggest the possibility to use bread as an innovative and inexpensive template for the development of highly-sustainable bone tissue engineering approaches.

## 1. Introduction

Bone substitution in critical-sized defects is still considered one of the greatest clinical challenges of our time [1,2,3,4,5]. Despite the latest progresses in bone tissue engineering (BTE) concerning both materials and manufacturing techniques, up to now, autologous bone is still considered the “gold standard” in grafting procedures due to its ability to support host bone healing and regeneration without triggering any adverse reaction, thus guaranteeing a good long-term outcome of the clinical treatment [6]. However, autologous bone grafting suffers from some limitations, such as donor site morbidity due to the need for performing at least two surgical procedures and low tissue availability [7,8]. Hence, it is necessary to keep on making investments on biomaterials research and new manufacturing technologies.

Bioactive glasses and glass-ceramics are considered promising materials for the production of scaffolds for BTE as an alternative therapeutic approach to autologous bone transplants [9,10,11]. Bioactive glasses, first introduced by Hench in 1969 [12,13,14,15], are strongly appreciated due to their ability to chemically bond to the host bone tissue by inducing the nucleation of an interfacial layer of nano-crystalline hydroxyapatite (HA) through an ion dissolution mechanism, which is able to trigger positive cell response in terms of adhesion, proliferation, differentiation and viability [16,17,18,19,20].

Among all the available manufacturing techniques used to process ceramics and glasses (e.g., solid free-form fabrication technologies, foaming, replication of sacrificial templates [21,22,23]), sponge replica method gained a foothold as one of the most effective and affordable strategies for the production of highly-reproducible and interconnected three-dimensional (3D) bone-like scaffolds able to mimic both healthy and pathologic bone [24]. The versatility of this method relies upon the possibility to use a wide variety of sacrificial templates and to synergistically combine the basic technique with several other approaches, including for instance electrospinning and gel-casting to mimic the Haversian canals [25] and to achieve a better control on both microstructure and mechanical properties [26,27]. Over time, several sacrificial templates have been employed, both of synthetic (e.g., commercial polymeric sponges [28]) and natural (e.g., marine sponges [29]) origin. Moreover, both traditional melt-derived glasses and sol-gel materials were successfully processed with promising and enthusiastic results [24,28,30,31].

At present polyurethane (PU) sponges, due to their easy availability, low cost and morphological similarity to trabecular bone in terms of open-cell architectures and high interconnectivity of the pores, are widely-used as sacrificial templates for the implementation of the method. The replication of PU foams for the production of glass-based bone substitutes was first introduced in 2006 by Chen and coworkers, who are considered the pioneers of this approach [28]. In general, the use of PU foams allows high porosity levels (~90 vol%) to be obtained; however, the mechanical properties of glass scaffolds are usually in the lower reference range considered for trabecular bone (<4 MPa) [29]. In order to overcome this drawback, usually high processing temperatures are used to obtain a better densification of the structure, even though high-temperature sintering is often associated to devitrification. The development of crystalline phases within the amorphous matrix can be responsible of a decrease in the bioactive potential of the system, which was for example assessed in the Hench’s 45S5 composition [32]; this is the main reason why other bioactive glasses with a large sintering window have been proposed over the last decade [33].

In recent years, researchers working in the biomedical field have taken inspiration from the study of natural structures [34,35] to improve and create highly mechanically and biologically performing devices. In particular, in order to achieve better mechanical properties, the choice of the sacrificial template plays a crucial role. Some research groups reported the advantages of using natural marine sponges to increase the mechanical properties of glass-based scaffolds [29,36]. Marine sponges, in fact, compared to commercial PU foams, are characterized by pore interconnectivity higher than 99% and porosity from 68 to 76 vol%, which is still acceptable for bone tissue engineering applications. Moreover, the presence of pores in the range of 150–500 µm allow new tissue ingrowth, while pores up to 200 µm induce the formation of an organized vascular network within the whole volume of the scaffold [29].

In 2011, Cunningham et al. [36] compared the performances of hydroxyapatite scaffolds derived from the replica of natural and synthetic sacrificial templates. The use of marine sponges allowed them to obtain compressive mechanical properties comparable to those of native spongy bone with pore size distribution ranging between 1–500 µm.

Marine sponges are not the only example of natural macroporous template used for this purpose. Among the other sacrificial templates of natural origin, corn stalks, mushrooms and cattail stem have been successfully used in combination with mesoporous bioactive glasses (MBGs) to produce multiscale macro-mesoporous scaffolds by sol-gel synthesis [37].

Inspired by all these approaches, we propose for the first time in this study the use of stale bread as a new sacrificial template for the production of macroporous glass scaffolds for bone tissue engineering. Bread is commonly considered a staple food for human consumption, and, as a result, it is available in a wide variety of types at a very low cost. In industrialized countries, bread is sold on the market also in packaged formats produced by standardized processes that minimize variation among different batches coming from the same company. In Italy and most of countries worldwide, packaged bread-based products must be sold with an expiry date, after which sale is not allowed anymore and expired bread becomes waste for disposal [38].

The driving force of this research activity was to combine biomaterials science and technology with waste management following a novel and creative approach. In fact, the aim was not only to explore the suitability of new macroporous templates (stale bread) for the production of bone repair scaffolds, but also to provide a contribute in saving wastes for the development of highly sustainable and eco-friendly strategies for BTE by minimizing costs and environmental impact. In the following sections, the production and characterization of bread-templated scaffolds will be reported and, in particular, morphological features, mechanical properties, and bioactive potential will be presented in detail.

## 2. Materials and Methods

### 2.1. Glass Preparation

47.5. B bioactive silicate glass with composition 47.5SiO_2_-20CaO-10MgO-2.5P_2_O_5_-10K_2_O-10Na_2_O (mol.%) [39] was used as a starting material for scaffold fabrication. The glass was produced by traditional melt-quenching route. Briefly, all the reagents (oxides and carbonates) were heated in a covered platinum crucible up to 1000 °C with a heating rate of 12 °C/min. After that, the platinum cap was removed and the temperature was increased up to 1500 °C with a heating rate of 15 °C/min. The melt was maintained at 1500 °C for 30 min and then poured in distilled water in order to obtain a glass frit. The frit was left to dry at room temperature overnight, crushed by ball milling (Pulverisette 0, Fritsch, Idar-Oberstein, Germany) and sieved to two different final grain sizes, below 25 and 32 µm (stainless steel sieves, Giuliani Technology Srl, Turin, Italy)

### 2.2. Scaffold Production

The traditional sponge replica method was properly revised in order to adapt the technique for using stale bread as a new template. Bread from industrial wastes (Roberto Industria Alimentare S.r.l., Treviso, Italy) was shaped (10 mm × 15 mm × 10 mm cuboids) and dried for 30 min in an oven at 80 °C in air in order to remove residual moisture. For the preparation of the slurry, a poly(vinyl alcohol) (PVA; Sigma-Aldrich, St. Louis, MO, USA) solution was prepared by dissolving PVA particles in water at 60 °C on a magnetic stirrer (200 rpm) until a transparent solution was obtained. After dissolution, evaporated water was replaced in order to restore the original PVA/H_2_O ratio. Then, glass powders were added by keeping the stirring rate constant until a homogeneous white slurry was obtained. Defining the proper slurry formulation was a crucial point in order to preserve the integrity of bread during the replica procedure while allowing the slurry to penetrate the whole porous volume of the template.

In a preliminary study, the slurry formulation was glass:PVA:water = 30:6:64 (wt%). An inefficient impregnation of the template, with the formation of an outer shell of glass which limited the penetration of the slurry to the inner core, was observed. In the attempt to solve this problem, slurry formulation was opportunely modified. Specifically, other three different slurry compositions were tested, obtained by varying the ratio of the slurry components and the size of the glass particles. The details are reported in Table 1.

The sacrificial template (bread blocks) was immersed into the slurry until complete impregnation was achieved. The green bodies were left to dry at room temperature for 48 h on a raised metallic grid in order to allow the exceeding slurry to drop down by gravity. Then, the greens were thermally treated at 750 °C for 3 h (heating rate 5 °C/min) to burn out the organic template and allow the strut to densify upon sintering.

### 2.3. Characterizations

#### 2.3.1. Differential Thermal Analysis (DTA)

Differential thermal analysis (DTA) was performed on 47.5B glass powder by using a DTA 404 PC instrument (Netzsch, Selb, Germany) in order to determine the characteristic temperatures of the glass; the temperature range was 20–1200 °C and the heating rate was 10 °C/min. Glass powders (100 mg) were introduced in Pt-Rh crucibles provided by the manufacturer; high-purity (≥99%) Al_2_O_3_ (100 mg) was used as a reference material.

#### 2.3.2. X-Ray Diffraction

X-Ray Diffraction analysis (XRD; 2θ within 10–70°) was performed on both as-quenched glass and crushed sintered scaffolds to detect the presence of crystalline phases. A X’Pert Pro PW3040/60 diffractometer (PANalytical, Eindhoven, The Netherlands) was used; parameters used for the measurement were: operating voltage 40 kV, filament current 30 mA, Bragg-Brentano camera geometry with Cu Kα incident radiation (wavelength λ = 0.15405 nm), step size 0.02°, and a fixed counting time per step of 1 s. Identification of crystalline phase was carried out by using X’Pert HighScore software 2.2b (PANalytical, Eindhoven, The Netherlands) equipped with the PCPDFWIN database (http://pcpdfwin.updatestar.com).

#### 2.3.3. In Vitro Bioactivity Tests

Simulated body fluid (SBF) was prepared according to the protocol reported by Kokubo and Takadama [40]. In vitro bioactivity tests were performed by immersing the scaffolds in SBF at 37 °C up to seven days in static conditions. A mass-to-volume ratio of 1.5 mg/ml was used, as suggested in previous studies [41,42]. The solution was completely replaced with fresh SBF every 48 h in order to simulate fluid circulation in physiological conditions and the pH was monitored in order to qualitatively evaluate the ionic exchange between the material and the solution. At the end of the experiment, the samples were rinsed with distilled water, dried overnight at 37 °C in incubator and stored in a sealed plastic box for further investigations.

#### 2.3.4. Porosity and Morphology

The porosity of the scaffolds was evaluated by density assessment according to Equation (1) [43]:

(1) $$\Pi =1-\frac{\rho_{s}}{\rho_{m}}\;$$

where Π is the total (fractional) porosity, ρ_s_ is the scaffold density and ρ_m_ is the density of the pore-free material. The density of the glass used in this study was calculated by Archimedes method and was found to be 2.64 g/cm^3^. The density of bread–templated scaffolds was calculated as the mass-to-volume ratio

The morphology of the scaffolds was investigated before and after in vitro bioactivity tests in SBF in order to evaluate pore features, pore interconnectivity and surface modification upon soaking. Moreover, preliminary morphological studies were performed on several bread sacrificial templates in order to choose the most suitable 3D architecture for the intended purpose. Morphological and compositional analyses were performed by a field-emission scanning electron microscope (FE-SEM; Supra^TM^ 40, Zeiss, Oberkochen, Germany) equipped with an energy dispersive spectroscopy (EDS) detector. Before being analyzed, both the scaffolds and the organic bread templates were sputter-coated with a conductive layer of chromium (~7 nm). The analysis was performed by using an accelerating voltage ranging between 5 and 15 kV.

#### 2.3.5. Mechanical Characterization

The compressive strength of scaffolds was evaluated through destructive crushing tests by using a MTS machine (QTest^TM^/10; cell load 2.5 kN, cross-head speed set at 0.5 mm/min). The failure stress was calculated as the ratio between the maximum load registered during the test and the resistant cross-sectional area, according to Equation (2):

(2) $$\sigma_{r}=\;\frac{F}{A}$$

where σ_r_ (MPa) is failure stress, F (N) is the maximum load and A (mm^2^) is cross-sectional area. The results were expressed as mean ± standard deviation assessed on five specimens, which were polished prior to the test by using SiC grit paper.

## 3. Results and Discussion

In this work, stale bread coming from industrial wastes was used as a novel sacrificial template to produce bioactive glass scaffolds by replication method. This well-known method, combined with the choice of the new template, exhibits attractive advantages including easy availability of the templating material, contribution to alimentary waste disposal, and transversal high sustainability and cost-effectiveness of the manufacturing process.

The suitability of a scaffold for BTE applications greatly relies upon its structural parameters such as total porosity, pore size and pore interconnectivity. These features have to be considered when producing a BTE scaffold, which should ideally exhibit total porosity between 50 and 90 vol%, pore size within 10–500 µm and inter-pore windows ≥100 µm [44].

**Figure 1:**
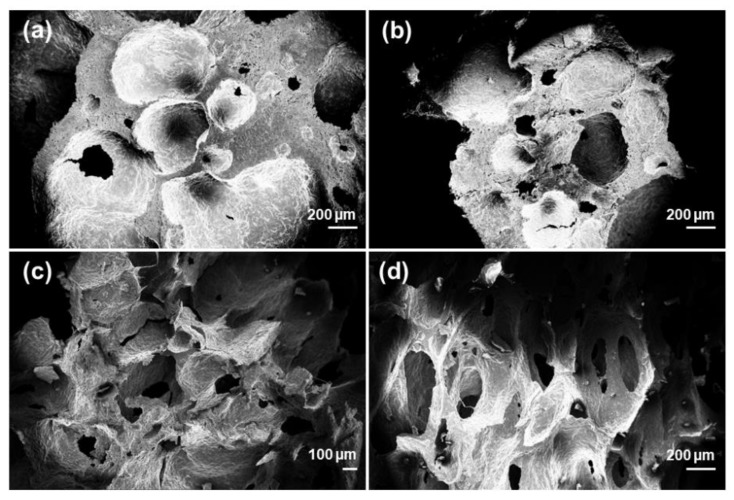
SEM micrographs of home-made bread (**a**,**b**) and industrial bread (**c**,**d**).

The characteristics of the template indeed dictate the properties of the final scaffold. Preliminary assessment of bread morphology by SEM revealed an open-cell porous architecture, with pore size and distribution potentially suitable for BTE scaffold production. However, it should be noticed that bread porosity is the result of a natural process, known as leavening, and, as a consequence, hardly controllable. In order to choose the template with the most regular and reproducible 3D architecture, SEM analysis was performed on several bread samples. SEM micrographs of home-made and industrial bread are shown in Figure 1. Home-made bread is characterized by a typical bubble-like morphology (Figure 1a,b), similar to that obtained by foaming methods [45], even if low inter-pore connectivity and irregular pore size (lack of reproducibility) make it unsuitable as scaffold template. Unlike rough bread, the industrial one—commercialized in packaged formats—revealed a trabecular-like morphology (Figure 1c,d) which was very similar to that of spongy bone. In this case, pore size and shape appeared to be more regular, homogeneously distributed and interconnected, Large macropores within 100–300 µm and smaller ones between 10–20 µm can be seen, which are definitely in the range mentioned above [44]. Therefore, commercial bread was selected as a sacrificial template for this study.

**Figure 2:**
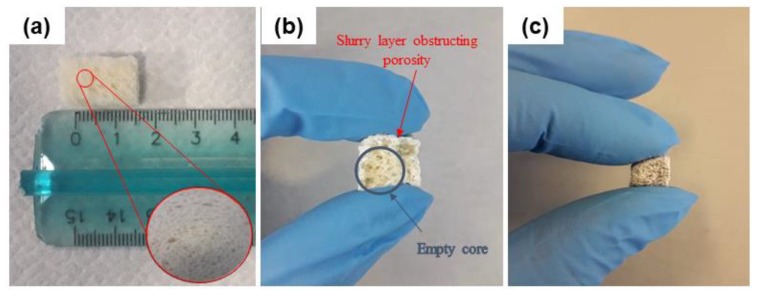
Bread macroporous template before impregnation (**a**); Green body obtained by using BDS_0 slurry composition (**b**) with inefficient template impregnation; green body belonging to the system BDS_a obtained by optimized procedure (**c**).

Sponge replica method was revised in order to adapt the process from traditional polymeric foams to our new organic template. In a traditional replication approach, the template is immersed into the slurry and then squeezed repeatedly to remove the excess suspension from the pores [28]. In this case, a crucial point was to retain the integrity of the template (Figure 2a) upon immersion, avoiding the collapse of the structure and the closure of the pores before sintering.

An extensive investigation was carried out to define and optimize the slurry formulation in order to allow the slurry penetrating inside the whole volume of the template. In this regard, the formulation BDS_0 was indeed unsatisfactory. As shown in Figure 2b, the slurry created a sort of shell around the bread cuboid. This was attributed to the high viscosity of the slurry, related to several factors including the high amount of PVA, the glass particles size and the relatively high amount of solid particles inside the composition.

Therefore, three different strategies were carried out in order to decrease the viscosity of the slurry. Considering the three components of the slurry, we acted on one parameter at a time to selectively study the effect of each of them on the final outcome. Specifically, we reduced PVA concentration, glass content and glass particle size for BDS_a, BDS_b, and BDS_c compositions, respectively (Table 1).

An example of BDS_a green body after drying is shown in Figure 2c: in this case, the slurry appeared to be homogeneously distributed in the whole volume of the bread cuboid and no obstruction of the peripheral pores was observed.

**Figure 3:**
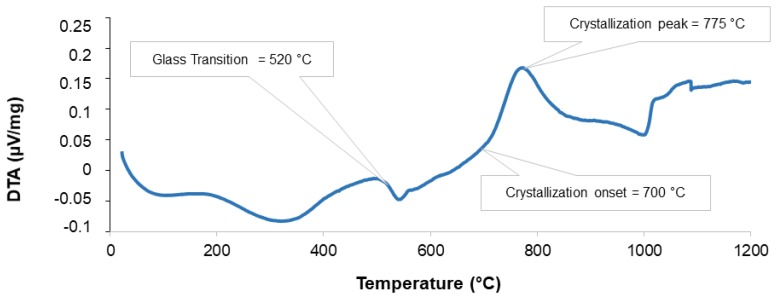
DTA thermograph of 47.5B bioactive glass and characteristic temperatures.

In order to select the best sintering temperature for obtaining mechanically resistant scaffolds without compromising bioactivity potential and HA-forming ability, DTA and XRD analyses were carried out. DTA curve of 47.5B glass is shown in Figure 3. The analysis revealed an exothermic peak at 775 °C, with the onset of crystallization detected at T_x_ = 700 °C and the glass transition temperature at T_g_ = 520 °C; the endothermic peak at around 1000 °C corresponds to the melting of the glass. These results are in good accordance with previous thermal data [46]. Despite the glass was characterized by a wide workability window (T_x_–T_g_), which potentially allows obtaining sintered scaffolds with a preserved amorphous nature, in this study, the sintering temperature was set at 750 °C, above the crystallization onset: this was due to the dramatic brittleness of scaffolds sintered at lower temperatures, as assessed in early trials.

**Figure 4:**
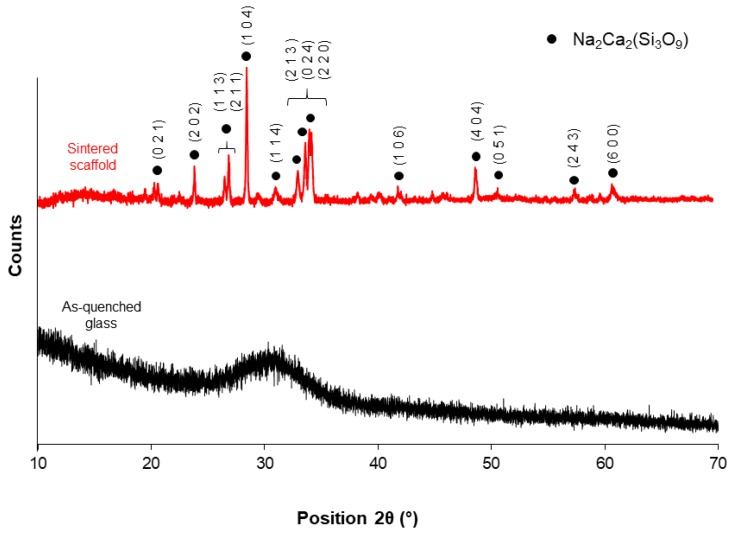
XRD patterns of as-quenched 47.5B bioactive glass (black) and sintered powdered scaffold (red).

XRD patterns of the as-quenched glass and powdered scaffold are reported in Figure 4. As expected, the pattern of the as-quenched material was characterized by an amorphous halo between 25–35°, which is typical of glassy silicate structures. On the contrary, the XRD pattern of the sintered scaffold exhibited sharp diffraction peaks associated to the devitrification of the system upon sintering, which is consistent with the results from thermal analysis. The sintering temperature was chosen in order to maximize the densification of the material and achieve adequate mechanical properties for BTE applications.

One crystalline phase was detected in the scaffold material (combeite, Na2Ca2(Si3O9), PDF code: 01-075-1686); this phase was also reported to form in a similar SiO_2_-CaO-Na_2_O-MgO-K_2_O-P_2_O_5_-based bioactive glass ceramic (CEL2) after thermal treatment above 950 °C [47] and, interestingly, was identified as the major phase of crystallized 45S5 Bioglass^®^ by some authors [48,49].

**Figure 5:**
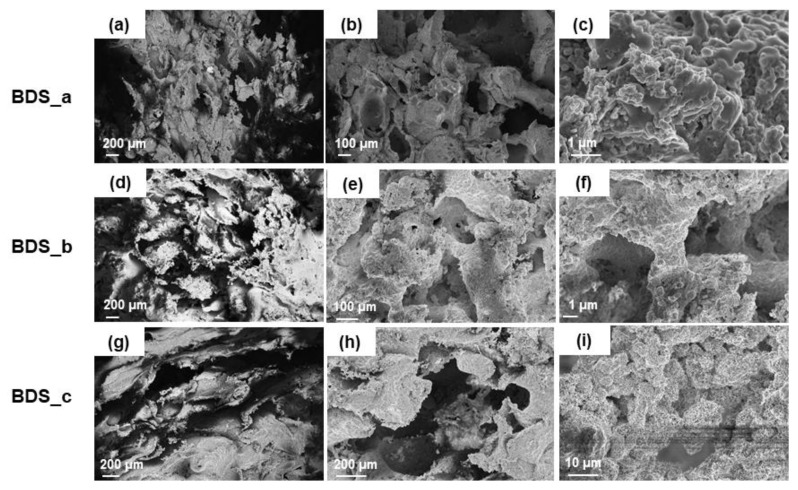
SEM morphological analyses performed on BDS_a (**a**–**c**), BDS_b (**d**–**f**), and BDS_c (**g**–**i**) scaffolds at different magnifications.

Figure 5 reports SEM images referred to the three different slurry formulations. It was found that, interestingly, the porosity of all the scaffolds was between 70–85 vol%, regardless of the manufacturing strategy used (BDS_a,b,c). This is an important achievement supporting the potential suitability of the scaffolds for BTE, since their porosity is definitely in the range of trabecular bone [43].

It is clear that, upon sintering, the device underwent remarkable modifications in terms of morphology and pore size distribution as compared to the original bread template. On the other hand, some significant differences can be pointed out among the three systems. Highly irregular surface and disorganized porosity were observed in BDS_b and BDS_c samples (Figure 5d,e,g,h, respectively). The 2D-view of the cross-section suggested the presence of voids in the structures, even if it was quite difficult to define the degree of interconnectivity between adjacent pores. It is possible that such voids were the result of a poor impregnation of the template, which is actually responsible for the absence of material in the core of the structure.

Reducing PVA amount was indeed found to be the most effective strategy to avoid the formation of an outer shell occluding the peripheral porosity. In this regard, the morphology of the starting template was still well recognizable in BDS_a samples shown in Figure 5a,b. Pore size was reduced as a consequence of the densification of the structure upon sintering, but it was still within the minimum required range. Inter-pore windows of 20–100 µm are potentially suitable to allow the vascularization of the graft [43].

At higher magnifications (Figure 5c,f,i), all the scaffolds belonging to the three systems showed the same wrinkled surface of the original template, which is good for the cell-device interactions. In fact, it is known that micrometric roughness on the implant surface can promote protein-mediated cell adhesion [50].

According to these observations, BDS_a system was chosen as the optimal composition for further investigations concerning mechanical compressive strength and in vitro bioactivity.

**Figure 6:**
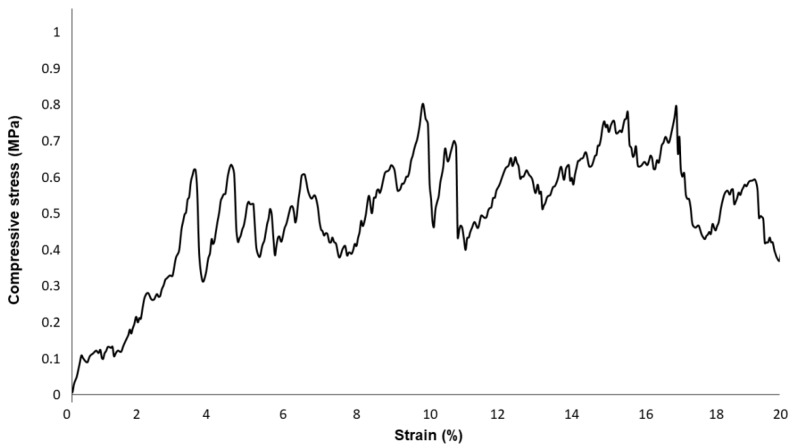
Typical stress-strain curve related to BDS_a scaffolds.

Mechanical characterization of the scaffolds obtained from BDS_a slurry, with total porosity of 72.0 ± 1.5 vol%, revealed a compressive strength of 0.62 ± 0.2 MPa, which is in the typical range of trabecular bone (0.1–16 MPa [44]), albeit close to the lower limit. Furthermore, the compressive strength of these bread-templated 47.5B scaffolds is about two times higher than that of PU-derived 45S5 Bioglass^®^ foams proposed by Chen et al. [28]. The curve reported in Figure 6 reveals the typical behavior of a cellular ceramic, where the multi-peak trend is related to multiple fracture events which occurred during the compression test [51].

**Figure 7:**
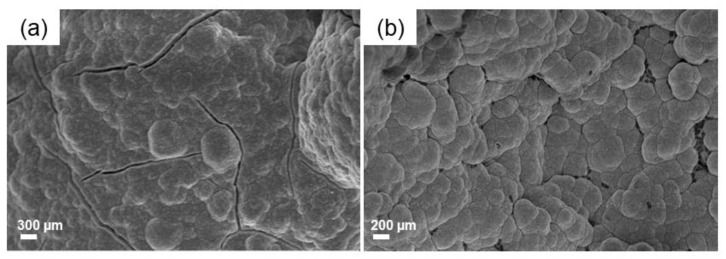
Surface modifications after immersion for 48 h (**a**) and one week (**b**) in SBF.

In vitro bioactivity tests up to seven days of immersion in SBF revealed an exceptional apatite-forming ability of the scaffold, in spite of devitrification that occurred upon sintering (Figure 4). After just 48 h, the scaffold surface appeared to be covered by a silica gel layer (with its typical cracks) on which calcium phosphate globular aggregates were already visible (Figure 7a); after one week, HA aggregates with typical cauliflower morphology completely covered the scaffold surface, thus suggesting the continuous evolution of the reaction layer as a result of the ion exchange between the scaffold and the SBF (Figure 7b).

**Figure 8:**
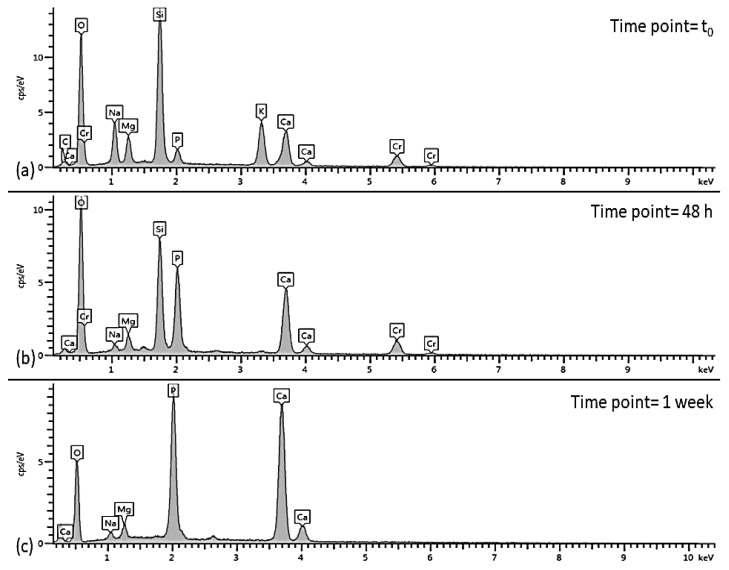
EDS spectra of the scaffold’s surface before in vitro bioactivity tests (**a**), after 48 h (**b**), and after one week-immersion in SBF (**c**).

Consistently to what observed by SEM morphological analyses, EDS assessments revealed an increase in the amount of Ca and P on the surface of the scaffold as compared to the original composition, which indicates the progressive growth of a calcium-phosphate layer at the interface between the scaffold and the fluid. After one-week immersion, the peak of Si disappeared from the EDS spectrum indicating that the scaffold surface has been completely covered by a thick layer of globular hydroxyapatite (Figure 8).

The Ca/P atomic ratio after 1-week immersion in SBF was around 0.5 ± 0.24 (measurements performed on three points), which is remarkably lower than the stoichiometric value of HA (1.67). Calcium-deficient HA was already observed on the surface of bioactive glasses with several compositions [52,53,54]. It is believed that a further increase of the Ca/P ratio is likely to be observed by extending the duration of the test up to 14–21 days, as already done in our previous work on 3D-printed glass scaffold with the same composition [55].

## 4. Conclusions

In this study, bread was proposed for the first time as a macroporous template for the production of BTE bioactive glass-derived scaffolds. Traditional foam replica method was opportunely modified in order to adapt the technique to the new organic template and slurry formulation was optimized in order to facilitate the complete impregnation of bread samples. Despite glass devitrification occurred upon heating, the sintering temperature selected allowed us to obtain suitable mechanical properties for trabecular bone applications while preserving the exceptional bioactivity of the system. Future studies deserve to be carried out to improve the repeatability and reliability of the method by a further optimization of the manufacturing process and sample preparation. Current results are promising and suggest the possibility to join the necessity to manage alimentary wastes with innovative and highly sustainable tissue engineering approaches.

## Figures and Tables

**Table 1:** Slurry compositions tested.

Name	Glass Particle Size (µm)	PVA (wt%)	H_2_O (wt%)	Glass (wt%)
BDS_0	≤32	6	64	30
BDS_a	≤32	1	69	30
BDS_b	≤32	6	69	25
BDS_c	≤25	6	64	30
